# Hydrops, Arthrogryposis, and Cerebellar Hypoplasia in a Fetus With a de Novo *BICD2* Variant: Expanding the Prenatal Phenotype of SMALED2B

**DOI:** 10.1002/pd.70024

**Published:** 2025-11-17

**Authors:** Francesca Romana Lepri, Ludovico Graziani, Lucia Menale, Milena Viggiano, Roberta Bucci, Angela Gentile, Leonardo Caforio, Antonio Novelli

**Affiliations:** ^1^ Laboratory of Medical Genetics Translational Cytogenomics Research Unit Bambino Gesù Children's Hospital IRCCS Rome Italy; ^2^ Genetics and Developmental Biology Unit Azienda Ospedaliera Universitaria Sassari Sassari Italy; ^3^ Obstetrics and Gynecology, Department of Neonatology Bambino Gesù Children's Hospital IRCCS Rome Italy; ^4^ Medical Genetics Unit, Department of Human Reproductive Medicine Bari Italy; ^5^ Università degli Studi di Milano Milan Italy

## Abstract

- What is already known about this topic?
  ◦ *BICD2* variants are associated with spinal muscular atrophy, lower extremity predominant type 2, typically diagnosed postnatally or after fetal demise.
  ◦ Prenatal presentations may include arthrogryposis multiplex congenita, hydrops, and fetal akinesia.
- What does this study add?
  ◦ This is the first report of a fetus with a prenatally detected *BICD2* variant showing increased nuchal translucency and hydrops on first‐trimester ultrasound examination.
  ◦ This is also the first prenatal case in which cerebellar hypoplasia has been documented by ultrasound in a disease related to the *BICD2* gene.

## 1 Case Presentation

A 37‐year‐old primigravida, pregnant after in vitro fertilization due to idiopathic infertility, was referred for a genetic consultation because of severe fetal hydrops at the second‐trimester ultrasound (US) examination. Preimplantation genetic testing (PGT) was not performed. The pregnancy began as a dichorionic twin gestation, with early intrauterine demise of one fetus. First‐trimester US at 12 + 5 gestational weeks (GWs) showed an increased nuchal translucency (NT) of 3.6 mm (CRL: 66.1 mm, > 99centile), absent nasal bone, bilateral pleural effusion, ascites, generalized subcutaneous edema, and reduced fetal movements. Additional findings included a single umbilical artery and tricuspid regurgitation. Maternal serologies for congenital infections were negative. The parents were nonconsanguineous; their family history was negative for genetic diseases and/or congenital malformations.

**FIGURE 1:**
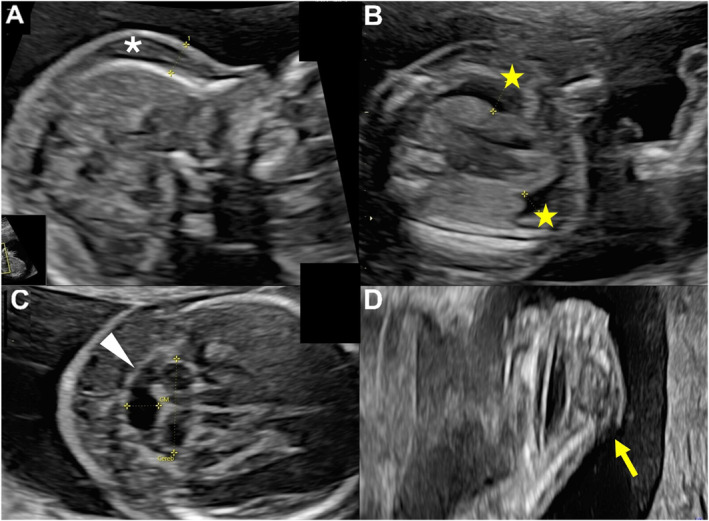
Second‐trimester ultrasound imaging. (A) Prefrontal edema measuring 6.4 mm (white asterisk). (B) Bilateral pleural effusion (yellow stars) measuring 6.3 and 4.56 mm respectively. (C) Transcerebellar diameter below 1% for gestational age (white arrowhead). (D) Congenital vertical talus (yellow arrow).

Further screening testing (e.g., NIPT) was not performed, and chorionic villus sampling (CVS) revealed normal male karyotype (46,XY) and chromosomal microarray (CMA) results. Subsequent targeted molecular panel for RASopathies (i.e., *BRAF, CBL, HRAS, KRAS, LZTR1, MAP2K1, MAP2K2, MRAS, NRAS, PPP1CB, PTPN11, RAF1, RASA2, RIT1, RRAS, RRAS2, SHOC2, SOS1*) was unremarkable. At 19 + 3 GWs, US examination revealed prefrontal edema (Figure 1A), persistent bilateral pleural effusion (Figure 1B), cerebellar hypoplasia with transverse cerebellar diameter reduced by 2.6 standard deviations (Figure 1C), and bilateral vertical talus (Figure 1D). The limbs appeared fixed in flexion, and fetal movements were decreased. Amniotic fluid was within the normal range (Table 1a).

**TABLE 1a:** Clinical data.

Case	Parental details			Gestation at diagnosis	Phenotypes (HPO terms)	Obstetric history	Family history	Outcome
1	Maternal	Age	37	Pregnancy previously terminated at 21w	*First trimester findings*: Increased NT, absent nasal bone, hydrops, decreased fetal movement *Second trimester findings*: Pleural effusion, cerebellar hypoplasia, AMC.	Gravida 1, para 0.	Unremarkable	Pregnancy termination, no autopsy.
		Medical history	Unremarkable					
	Paternal	Age	36					
		Medical history	Unremarkable					

Abbreviations: AMC, Arthrogryposis multiplex congenita; NT, Nuchal Translucency.

Due to the evolving phenotype, a trio‐based exome sequencing (ES) was performed using DNA from chorionic villi and parental blood. ES identified a de novo heterozygous novel missense variant in *BICD2* (NM_001003800.2:c.553 G>C; p. (Glu185Gln)). It was classified as likely pathogenic (C4) following ACMG/AMP criteria (Table 1b). After genetic counseling, the couple opted for termination of pregnancy at 21 weeks. No fetal autopsy was performed.

**TABLE 1b:** Genetic findings.

Procedure (Gest. Age)	Direct/culture?	Performed test	Gene (name; REFSEQ)	Known disease (OMIM)	Variant	ACMG classification	Criteria applied	Inheritance & zygosity	Interpretation
CVS (12 + 5)	Cultured	Trio exome sequencing	*BICD2*	SMALED2 (#615290, 618291)	NM_001003800.2:c.553 G>C; p. (Glu185Gln)	C4	PS2_Strong, PM1_Moderate, PM2_Supporting, PP3_Supporting	de novo	Causative of fetal phenotype

Abbreviations: CVS, Chorionic villus sampling; SMALED2, Spinal muscular atrophy, lower extremity‐predominant, 2, autosomal dominant.

## 2 Discussion

Heterozygous variants in *BICD2* are associated with autosomal dominant spinal muscular atrophy with lower extremity predominance (SMALED2), which encompasses a milder, postnatal‐onset form (SMALED2A) and a more severe prenatal‐onset phenotype (SMALED2B), typically presenting with fetal akinesia, multiple congenital contractures, and adverse outcomes [1]. Although most cases of SMALED2B have been identified postnatally or after fetal demise, increasing application of ES in prenatal settings has led to the recognition of consistent antenatal features [2, 3].

In this report, prenatal ES identified a likely pathogenic variant in *BICD2*, enabling a definitive diagnosis of SMALED2B with expanded phenotypic features. Notably, signs such as increased NT, hydrops, and reduced fetal movements were already evident in the first trimester, preceding the suspicion of arthrogryposis multiplex congenita (AMC). By the second trimester, additional findings, including fixed limb postures, bilateral vertical talus, cerebellar hypoplasia, and absent fetal movements, further supported the hypothesis of a neuromuscular disorder.

Recent literature has described fetuses with *BICD2* missense variants presenting with overlapping features, including severe AMC, pterygia, second‐trimester fetal hydrops, and early akinesia [4, 5]. These cases collectively suggest a consistent prenatal phenotype that, although variable in expressivity, can now be increasingly recognized on mid‐trimester ultrasound, especially in association with de novo variants in the coiled‐coil domains of *BICD2*. However, none of the previously reported cases described increased NT, hydrops, or reduced fetal movements as early as the first trimester. This highlights the possibility that *BICD2*‐related disease may be suspected and diagnosed much earlier, prenatally.

The cerebellar hypoplasia observed in our case may also reflect an emerging neurodevelopmental component of *BICD2*‐related disorders. Experimental data and human studies have proposed that disruption of Rab6A–BICD2 interaction impairs neuronal migration, potentially contributing to cerebellar and cortical malformations [6]. In the presence of posterior fossa anomalies, fetal brain MRI, most commonly performed between 20 and 24 weeks, may provide additional information on cerebellar and vermian morphology. To date, no prenatal brain anomalies have been reported in association with *BICD2* variants, whereas postnatal cases have included cerebellar hypoplasia and lissencephaly [6]. Importantly, this is the first report to document cerebellar hypoplasia on prenatal US imaging in a fetus with a de novo *BICD2* variant, expanding the known phenotype and suggesting that neurological involvement may be under‐recognized in this condition.

Overall, this case expands the prenatal phenotype of *BICD2*‐associated disorders to include cerebellar hypoplasia and supports the inclusion of ES in the diagnostic workup of fetuses with nonimmune hydrops, increased NT, or suspected AMC, particularly when first‐tier tests such as chromosome analysis, CMA, and targeted panels are inconclusive. Moreover, our findings are consistent with the growing consensus that broad‐based sequencing approaches, including ES, should increasingly be considered as first‐tier tests in high‐risk prenatal cases, as a stepwise approach may delay diagnosis and impact parental decision‐making.

## Funding

The authors have nothing to report.

## Ethics Statement

The authors have nothing to report.

## Consent

Informed consent was obtained for all diagnostics, and written informed consent was secured from the patients to publish medical data, including pictures, following the Declaration of Helsinki.

## Conflicts of Interest

The authors declare no conflicts of interest.

## Data Availability

The data that support the findings of this study are available from the corresponding author upon reasonable request.
